# EPHB4 inhibition activates ER stress to promote immunogenic cell death of prostate cancer cells

**DOI:** 10.1038/s41419-019-2042-y

**Published:** 2019-10-22

**Authors:** Vinay Sagar, Rajita Vatapalli, Barbara Lysy, Sahithi Pamarthy, Jonathan F. Anker, Yara Rodriguez, Huiying Han, Kenji Unno, Walter M. Stadler, William J. Catalona, Maha Hussain, Parkash S. Gill, Sarki A. Abdulkadir

**Affiliations:** 1 Department of Urology, The Robert H. Lurie Comprehensive Cancer Center, Northwestern University Feinberg School of Medicine, Chicago, IL 60611 USA; 2 Atrin Pharmaceuticals, Pennsylvania Biotechnology Center, Doylestown, PA 18902 USA; 3 Department of Medicine, Section of Hematology/Oncology, University of Chicago, Chicago, IL 60637 USA; 4 Department of Urology and Medical Social Sciences (DEV), Northwestern University Feinberg School of Medicine, Chicago, IL 60611 USA; 5 Division of Hematology/Oncology, Department of Medicine, Northwestern University Feinberg School of Medicine, Chicago, IL 60611 USA; 6 Division of Hematology, Department of Medicine, USC Norris Comprehensive Cancer Center, Keck School of Medicine, University of Southern California, Los Angeles, CA 90033 USA; 7 Department of Pathology, Northwestern University Feinberg School of Medicine, Chicago, IL 60611 USA

**Keywords:** Targeted therapies, Prostate cancer, Apoptosis

## Abstract

The EPHB4 receptor is implicated in the development of several epithelial tumors and is a promising therapeutic target, including in prostate tumors in which EPHB4 is overexpressed and promotes tumorigenicity. Here, we show that high expression of EPHB4 correlated with poor survival in prostate cancer patients and EPHB4 inhibition induced cell death in both hormone sensitive and castration-resistant prostate cancer cells. EPHB4 inhibition reduced expression of the glucose transporter, GLUT3, impaired glucose uptake, and reduced cellular ATP levels. This was associated with the activation of endoplasmic reticulum stress and tumor cell death with features of immunogenic cell death (ICD), including phosphorylation of eIF2α, increased cell surface calreticulin levels, and release of HMGB1 and ATP. The changes in tumor cell metabolism after EPHB4 inhibition were associated with MYC downregulation, likely mediated by the SRC/p38 MAPK/4EBP1 signaling cascade, known to impair cap-dependent translation. Together, our study indicates a role for EPHB4 inhibition in the induction of immunogenic cell death with implication for prostate cancer therapy.

## Introduction

Prostate cancer (PC) is the most common cancer in men worldwide. An estimated 174,650 new cases and 31,620 new deaths will be reported in the United States in 2019^1^. While therapy targeted at the androgen receptor pathway shows initial efficacy, a majority of patients with advanced PC go on to develop resistance^2–4^. Thus, there is a need for novel therapies that target additional pathways important for the growth and survival of prostate cancer cells. For example, tyrosine kinase inhibitors (TKIs) are being investigated as an additional strategy to treat prostate cancer^5–8^. Receptor tyrosine kinases (RTKs) regulate of cell proliferation, migration, apoptosis, and survival, and plays a role in the progression and development of multiple cancers^9,10^. Targeting RTKs enzymatic activity is emerging as a therapeutic strategy in many cancer. However, this approach is facing some constraints due to development resistance against these inhibitors^11,12^. Combination therapy approaches have to be developed to overcome this problem.

EPHB4 receptor tyrosine kinase is involved in many cellular processes such as cell growth, survival, angiogenesis, vasculogenesis, and neural development^13^. EPHB4 specifically binds to Ephrin B2 ligand and evokes bidirectional signaling^13^. Several reports have shown EPHB4 overexpression in various cancers including those of the prostate, breast, colon, head and neck, and lung^14–18^. Xi et al.^19^ showed that EPHB4 expression is increased in 66% of prostate tumor samples. EPHB4 is induced by the key molecular alterations implicated in prostate cancer and castrate-resistant prostate cancer development, including loss of tumor suppressors *PTEN* and *TP53*, and activation of the PI3K pathway^19^. Another report demonstrated that EPHB4 knockdown by siRNA resulted in a decrease in invasion and migration of prostate cancer cell lines^20^. However, neither the underlying mechanisms nor the functional consequences of EPHB4 inhibition in advanced prostate cancer are well understood. Here, we investigate the mechanisms by which EPHB4 inhibition affects cell viability. EPHB4 receptor inhibition in PC cells inhibited a SRC/MAPK/MYC pathways leading to reduced glucose transporter GLUT3 levels, decreased glucose uptake and intracellular ATP levels. Consequently, cells showed elevated ER stress and death with features of immunogenic cell death (ICD).

## Results

### High EPHB4 expression correlates with advanced prostate cancer stage and poor outcome

**Fig. 1:**
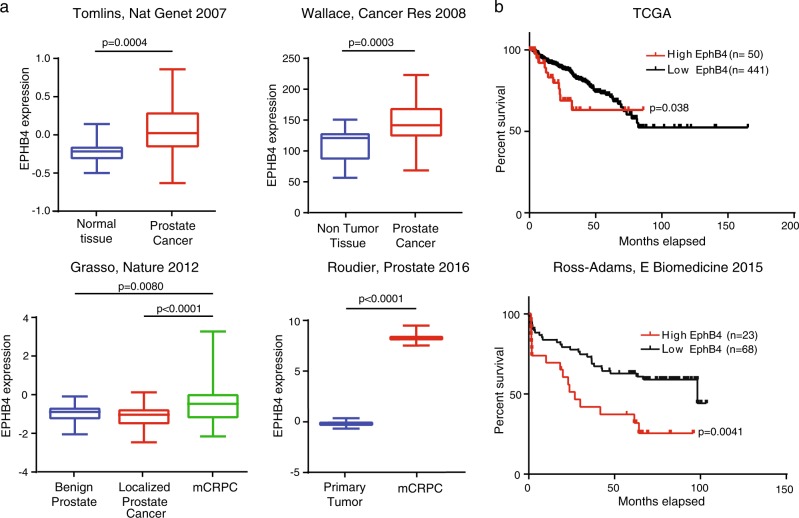
EPHB4 is overexpressed in advanced prostate and is associated with poor outcome. **a** EPHB4 expression was analyzed in different published datasets as indicated: Grasso, Nature 2012; Tomlins, Nat Genet 2007; Wallace, Cancer Res 2008; and Roudier, Prostate 2016. **b** Kaplan–Meier Survival analysis of TCGA Provisional prostate and CamCap (EbioMedicine, 2015) datasets for high EPHB4 and low EPHB4 expression

Previous studies have shown that EPHB4 promotes tumor cell survival and is overexpressed in prostate cancer^19,20^. We examined *EPHB4* mRNA expression in published human prostate cancer expression datasets and observed higher *EPHB4* expression in metastatic castration-resistant prostate cancer (mCRPC) as compared to the corresponding normal or primary tumor tissues (Fig. 1a). We also observe a positive correlation between EPHB4 expression levels and biochemical relapse-free survival in both the Cancer Genome Atlas (TCGA) and Ross-Adams^21^ datasets (Fig. 1b). Collectively, these results indicate that EPHB4 is a valuable prognostic biomarker and raises the hypothesis that it could be a therapeutic target in prostate cancer patients.

### EPHB4 inhibition decreases cell viability and induces apoptosis

**Fig. 2:**
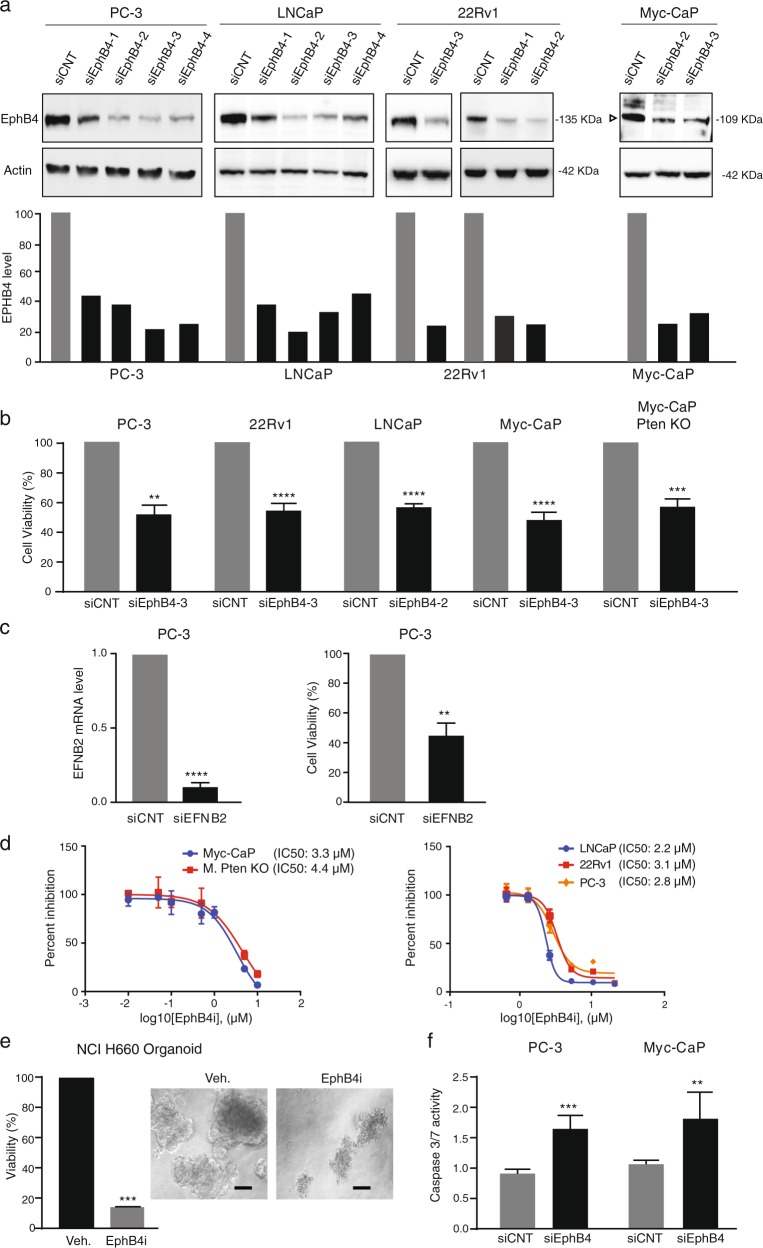
EPHB4 decreases cell viability and induces apoptosis. **a** EPHB4 knockdown efficiency was analyzed by western analysis after 72 h transfection of EPHB4 siRNAs or non-targeted siRNA in all prostate cancer cell lines. Open triangle indicate specific bands. **b** Prostate cancer cell lines were transfected with EPHB4 siRNA or scrambled siRNA for 72 h and cell viability determined using MTS assay. Experiments were performed in triplicate (*n* = 3). **c** PC-3 cells were transfected with EFNB2 siRNA or non-targeting siRNA for 72 h and cell viability determined using MTS assay and knockdown efficiency was analyzed at mRNA level by qRT-PCR. Experiments were performed in triplicate (*n* = 3). **d** Dose response of Myc-CaP, Myc-CaP Pten KO and LNCaP were assessed by CCK8 assay and, PC-3 and 22Rv1 cell viability by MTS assay after treatment with EPHB4i (NVP-BHG712 or DMSO) for 72 h. Data are presented as mean ± standard deviation (*n* = 3) from three different independent experiments. **e** NCI-H660 cell organoids were established for 1 week, then treated with EPHB4 inhibitor, NVP-BHG712 (EphB4i) 3 µM or DMSO for 1 week. Viability of organoids were measured by Celltitre 3D Glo assay. Organoid images were visualized at 20X and scale bar represents 100 μm. Data are presented as mean ± standard deviation (*n* = 3) from three different independent experiments. **f** PC-3 and Myc-CaP cells were transfected with EphB4 siRNA or non-targeted siRNA for 72 h and cell viability and caspase activity was determined by Caspase-Glo 3/7 assay. Data are presented as mean ± standard deviation (*n* = 3) from three different independent experiments. Statistical significance was analyzed by two-tailed Student’s *t*-test (each treated group is compared to control group). *P < 0.05, **P < 0.01, ***P < 0.001, ****P < 0.0001

To determine the functional significance of EPHB4 overexpression in prostate cancer, we knocked down *EPHB4* with specific siRNAs targeting EPHB4 (Fig. 2a). EPHB4 knockdown in human (PC-3, 22Rv1 and LNCaP) and mouse (Myc-CaP & Myc-CaP; Pten KO) prostate cancer cell lines resulted in a decrease in cell viability measured after 72 h (Fig. 2b). Similar results were seen after treatment with small molecule EPHB4 inhibitor, NVP-BHG712 on the viability of Myc-CaP, Myc-CaP Pten KO, PC-3, 22Rv1, and LNCaP cells (Fig. 2d). In addition we observed similar effects after the knockdown of EPHB4 ligand EFNB2 (Fig. 2c). We next examined the effects of EPHB4i on the viability of organoids generated from the neuroendocrine prostate cancer cell line, NCI-H660, and found a decrease in organoid viability and size after EPHB4 (Fig. 2e). The reduced viability caused by EPHB4 inhibition occurred through apoptosis, as indicated by increased caspase-3/7 activation (Fig. 2f). Collectively, our results show that inhibition of the EPHB4 receptor or its ligand EFNB2 decreases cell viability and induces apoptosis in prostate cancer cells.

### EPHB4 inhibition induces immunogenic cell death

**Fig. 3:**
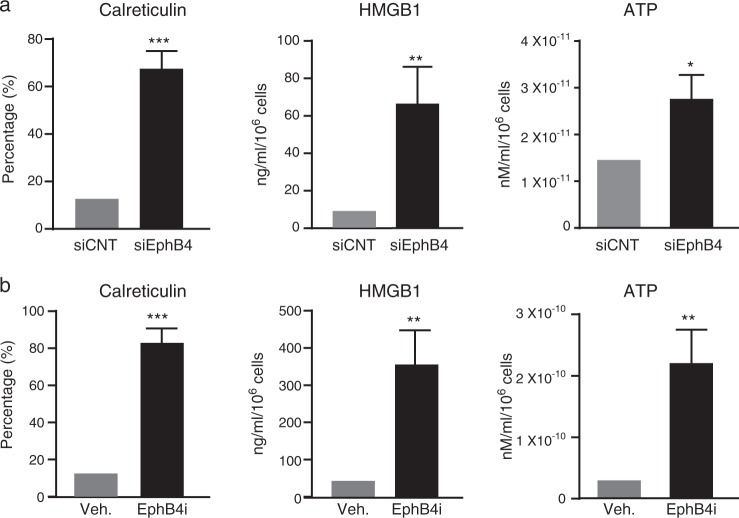
EPHB4 inhibition induces immunogenic cell death. **a** ICD was assessed in vitro from Myc-CaP cells transfected with siEphB4 or siCNT for 72 h. **b** Myc-CaP cells were treated with EphB4 inhibitor, NVP BHG712 (3 µM, EPHB4i), or DMSO (Veh.) for 72 h. ICD markers were assessed as follows: Calreticulin (flow cytometry), HMGB1 (ELISA), and ATP (luminescence assay) in biological triplicates, technical duplicates, compared to siCNT. Data are presented as mean ± standard deviation (*n* = 3) from three different independent experiments. Statistical significance was analyzed by two-tailed Student’s *t*-test (each treated group is compared to control group). *P < 0.05, **P < 0.01, ***P < 0.001, ****P < 0.0001

Some therapeutic agents are known to cause cell death by immunogenic cell death that can be exploited in immunotherapy. We examined whether EPHB4 inhibition induced immunogenic cell death (ICD) by assessing changes in the hallmarks of ICD, including cell surface levels of calreticulin, non-histone nuclear protein high-mobility group box 1 (HMGB1) release, and ATP release from Myc-CaP cells transfected with control (siCNT) or EPHB4-targeting siRNA (siEphB4–3). EphB4 inhibition resulted in increased cell surface localization of calreticulin and the release of HMGB1 and ATP into the extracellular space (Fig. 3a). Similar results were obtained in Myc-CaP cells treated with NVP-BHG712 (EphB4i) compared to Vehicle (Veh) (Fig. 3b). These results indicate that EPHB4 inhibition induces cell death consistent with ICD.

### EPHB4 inhibition induces endoplasmic reticulum stress through metabolic changes

**Fig. 4:**
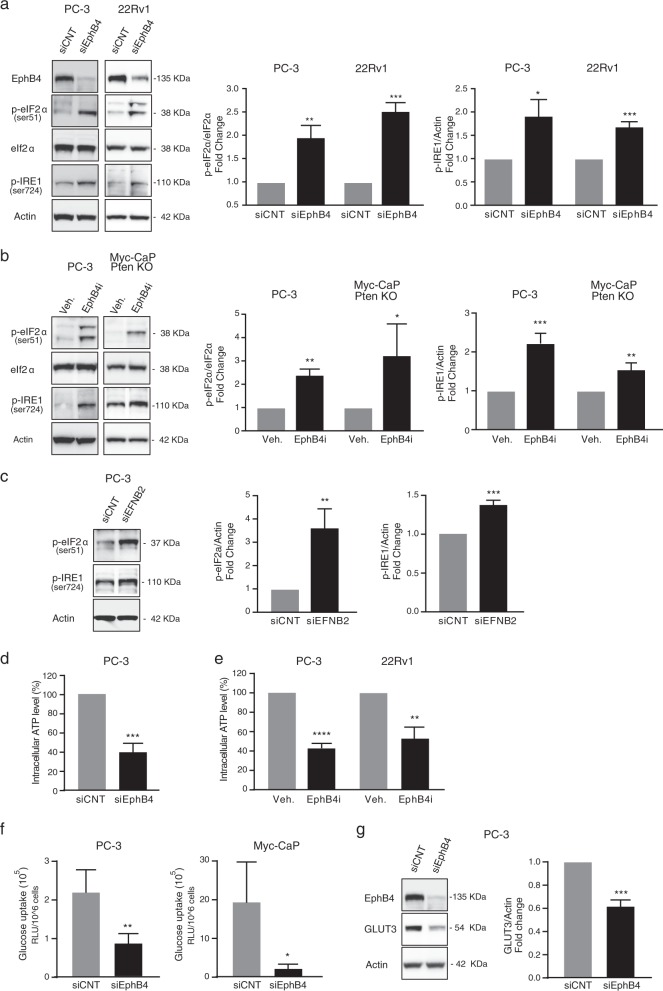
EPHB4 inhibition induces ER stress through ATP depletion and decreases glucose uptake and glucose transporter. **a** Western analysis with indicated antibodies on extracts from PC-3 and 22Rv1 cells transfected with control (siCNT) or with EphB4-specific siRNA (siEphB4) after 72 h. Data are presented as mean ± standard deviation from atleast three different independent experiments. **b** PC-3 and Myc-CaP;Pten KO cells were treated with 3 µM of EphB4i or Vehicle (DMSO) for 72 h, and the total extract was analyzed by western blot with indicated antibodies. Data are presented as mean ± standard deviation (*n* = 3) from three different independent experiments for p-eIF2α and p-IRE1 blot. Statistical significance was analyzed by two-tailed Student’s *t*-test (treated group is compared to control group). **c** Western blots with indicated antibodies on extracts from PC-3 cells transfected with control (siCNT) or with EphrinB2-specific siRNA (siEFNB2) after 72 h. Data are presented as mean ± standard deviation (*n* = 3) from three different independent experiments. **d**, **e** Intracellular ATP level was measured in PC-3 cells transfected with siEphB4 or EphB4 inhibitor and 22Rv1 cells treated with EphB4 inhibitor using PerkinElmer ATP lite assay. Data are presented as mean ± standard deviation (*n* = 3) from three different independent experiments. **f** Glucose uptake was measured in PC-3 and Myc-CaP cells transfected with siEphB4 or siCNT for 72 h using Promega glucose uptake Glo kit. Data are presented as mean ± standard deviation (*n* = 3) from three different independent experiments. **g** Western blot on extracts from PC-3 cells transfected with control (siCNT) or with EphB4-specific siRNA (siEphB4) after 72 h with indicated primary antibodies. Data are presented as mean ± standard deviation (*n* = 3) from three different independent experiments. Statistical significance was analyzed by two-tailed Student’s *t*-test (treated group is compared to control group). *P < 0.05, **P < 0.01, ***P < 0.001, ****P < 0.0001

Previous studies have suggested that endoplasmic reticulum (ER) stress plays a major role in intracellular signaling pathways that induce ICD^22,23^. The ER stress response is initiated through phosphorylation of eukaryotic translation initiation factor 2α (eIF2α)^24^, which has been proposed as a characteristic marker for ICD^24–26^. We therefore analyzed the phosphorylation status of eIF2α in PC-3 and 22Rv1 cells upon EPHB4 knockdown by siRNA (siEPHB4–3 or −4) compared to control siRNA (siCNT). EPHB4 inhibition increased phosphorylation of eIF2α at serine 51 (Fig. 4a). Similar results were seen in PC-3 and Myc-CaP;Pten KO cells treated with small molecule EPHB4 inhibitor (EPHB4i) (Fig. 4b). In addition to eIF2α, inositol-requiring enzyme 1 (IRE1) represents another ER protein that serves as ER stress sensor and mediates the unfolded protein response (UPR)^27,28^. Like eIF2α activation, IRE1 phosphorylation increased after EPHB4 knockdown by EPHB4-specific siRNA in PC-3 and 22Rv1 cells (Fig. 4a). Similar results were observed in PC-3 and Myc-CaP;Pten KO cells treated with EphB4i compared to vehicle control (Veh.) (Fig. 4b). To investigate whether EPHB4 mediated effect on ER stress is ligand-dependent, we analyzed the effect of EPHB4 ligand -EFNB2 knockdown on phosphorylation of eIF2α at serine 51 and IRE1 at serine 724. Consistent with our observations above, inhibition of EFNB2 resulted in a similar increase in phosphorylation of eIF2α at serine 51 and IRE1 at serine 724 in PC-3 cells (Fig. 4c). These data indicate that EPHB4 inhibition triggers the ER stress response in prostate cancer cells, which is associated with ICD. Cellular processes in the ER require energy in the form of ATP and perturbation of these processes induce ER stress^29^. Therefore, we sought to determine whether the ER stress induced by EPHB4 inhibition is related to ATP depletion in cancer cells. We measured the intracellular ATP level after EPHB4 inhibition in PC-3 cells transfected with siEPHB4–3 or siCNT. The levels of intracellular ATP were significantly lower in siEPHB4-transfected PC-3 cells (Fig. 4d). Similar results were obtained with EPHB4i-treated PC-3 and 22Rv1 cells (Fig. 4e). These results point to a decline in cellular ATP levels upon EPHB4 inhibition as a possible cause of ER stress.

The ER depends on extrinsic energy sources via oxidative phosphorylation or glycolysis^30,31^. We tested whether the decrease in intracellular ATP was associated with changes in glucose metabolism. The first essential step in glucose metabolism is glucose uptake. Both PC-3 and Myc-CaP cells demonstrated significantly reduced glucose uptake after EPHB4 knockdown by siEPHB4–3 (Fig. 4f). EPHB4 knockdown mediated decline in glucose uptake could be due to several steps of metabolic dysregulation including reduced glucose transport. To assess the impact of EPHB4, we monitored the levels of glucose transporters, in response to EphB4 knockdown. We observed that EPHB4 depletion in PC-3 cells resulted in a significant decrease in GLUT3 protein levels (Fig. 4g). Collectively, these data suggest that EPHB4 inhibition induces ER stress through ATP deficiency, arises due to decrease levels of glucose transporter, leading to decreased glucose uptake.

### EPHB4 inhibition decreases c-MYC level and P38 MAPK pathway

**Fig. 5:**
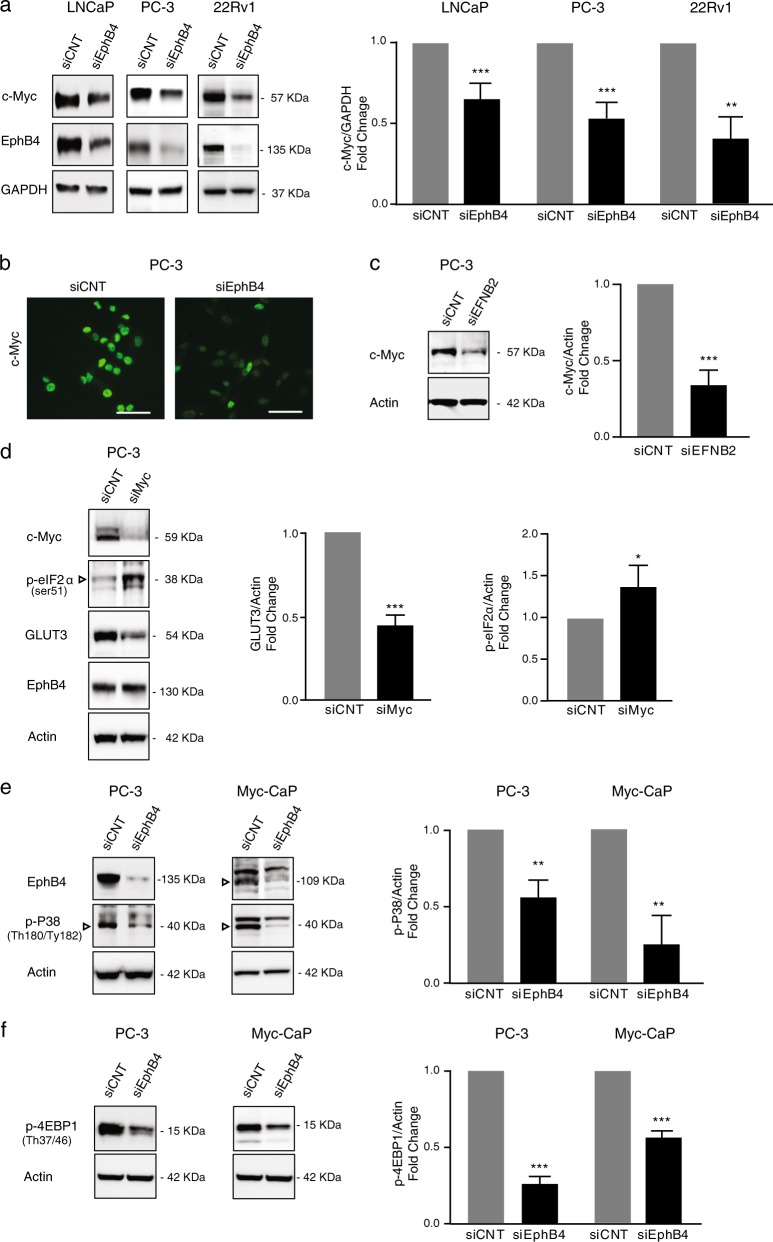
EPHB4 inhibition decreases Myc and phosphorylation of p38 MAPK and 4EBP1. **a** Western blot with indicated antibodies on LNCaP, PC-3, and 22Rv1 cells transfected with the indicated siRNAs for 72 h. Data are presented as mean ± standard deviation (*n* = 3) from three different independent experiments. Statistical significance was analyzed by two-tailed Student’s *t*-test (treated group is compared to control group). *P < 0.05, **P < 0.01, ***P < 0.001, ****P < 0.0001. **b** c-MYC expression was analyzed by Immunofluorescence in PC-3 cells transfected with indicated siRNAs for 72 h. Image was visualized at ×40 and scale bar represents 50 μm. **c** Western blots with indicated antibodies on extracts from PC-3 cells transfected with control (siCNT) or with EphrinB2-specific siRNA (siEFNB2) after 72 h. Data are presented as mean ± standard deviation (*n* = 3) from three different independent experiments. **d** PC-3 cells were transfected with siMYC or siCNT for 72 h and p-eIF2a at ser51, GLUT3, and EPHB4 level were measured by western blot. Data are presented as mean ± standard deviation (*n* = 3) from three different independent experiments. **e**, **f** Western blots with indicated antibodies on extracts from PC-3 and Myc-CaP cells transfected with control (siCNT) or with EphB4-specific siRNA (siEphB4) after 72 h. Data are presented as mean ± standard deviation from atleast three different independent experiments. Statistical significance was analyzed by two-tailed Student’s *t*-test (treated group is compared to control group). *P < 0.05, **P < 0.01, ***P < 0.001, ****P < 0.0001. Open triangles indicate specific bands

The c-MYC oncoprotein, a known regulator of glucose metabolism and has previously been linked to EPHB4 in colon carcinoma cells^32–34^. Therefore, we postulated that the decrease in the glucose transporter and glucose uptake after EPHB4 inhibition could be a result of changes in MYC levels. We therefore quantified MYC at protein level and expression after EPHB4 knockdown by siRNA (siEPHB4–3) compared to control siRNA (siCNT). EPHB4 depletion decreased c-MYC protein in LNCaP, PC-3 and 22Rv1 cells (Fig. 5a). In addition, we also observed decreased MYC protein expression after EPHB4 knockdown in PC-3 cells by immunofluorescence staining (Fig. 5b). Similar to EPHB4 inhibition, we observed a decreased in MYC protein in PC-3 cells transfected with siEFNB2 (Fig. 5c). Thus decreased MYC levels after EPHB4 inhibition may lead to decreased GLUT3 expression. To show this directly, we analyzed the effect of MYC inhibition on GLUT3 levels in PC-3 cells. As shown in Fig. 5d, we observed that MYC inhibition by siRNA (siMYC) decreased GLUT3 expression and no change in the level of EPHB4 in PC-3 cells. In addition, we also analyzed the phosphorylation status of eIF2α at Ser51 in PC-3 cells upon MYC knockdown by siRNA and observed an increased phosphorylation of eiF2α at Ser51 (Fig. 5d). These results suggest that EPHB4 inhibition leads to decrease in GLUT3 expression and phosphorylation of eIF2α through MYC downregulation.

How EPHB4 inhibition may affect MYC levels is not known. Previous reports have demonstrated that c-MYC expression is controlled by multiple signaling pathways including the MAPK pathway^35–37^, while EPHB4 is linked to MAPK signaling in a context-dependent manner^38^. To investigate whether MAPK signal transduction pathways are involved in MYC downregulation after EPHB4 inhibition, we analyzed the phosphorylation of p38 MAPK in PC-3 and Myc-CaP cells upon EPHB4 knockdown by siRNA (siEPHB4–2 or −3) compared to control siRNA (siCNT). We observed that the phosphorylation of p38 MAPK at Thr180/Tyr182 decreased after EPHB4 knockdown in both cell lines (Fig. 5e). As a result, we hypothesized that decrease in phosphorylation of p38 MAPK leads to decrease in phosphorylation of 4EBP1, which is known as translation repressor protein. 4EBP1 regulates the cap-dependent translation of many oncogenic proteins, including MYC, via eukaryotic translation initiation complex eIF4E^39,40^. When hypophosphorylated, 4EBP1 binds to eIF4E and blocks cap-dependent translation. Thus phosphorylation of 4EBP1 which is induced by p38 MAPK leads to an increase in cap-dependent translation^40,41^. We next examined the phosphorylation status of 4EBP1 upon EPHB4 knockdown by siRNA (siEPHB4–3) compared to control siRNA (siCNT). As expected, EPHB4 inhibition decreased phosphorylation of 4EBP1 at Thr37/46 in PC-3 and Myc-CaP cells (Fig. 5f). Collectively, these data suggest that EPHB4 inhibition decreases the levels of glucose transporter, leading to decreased glucose uptake, as well as metabolic perturbations through MYC modulation which is regulated by p38 MAPK-4EBP1.

### EPHB4 regulates p38 MAPK through SRC kinase

**Fig. 6:**
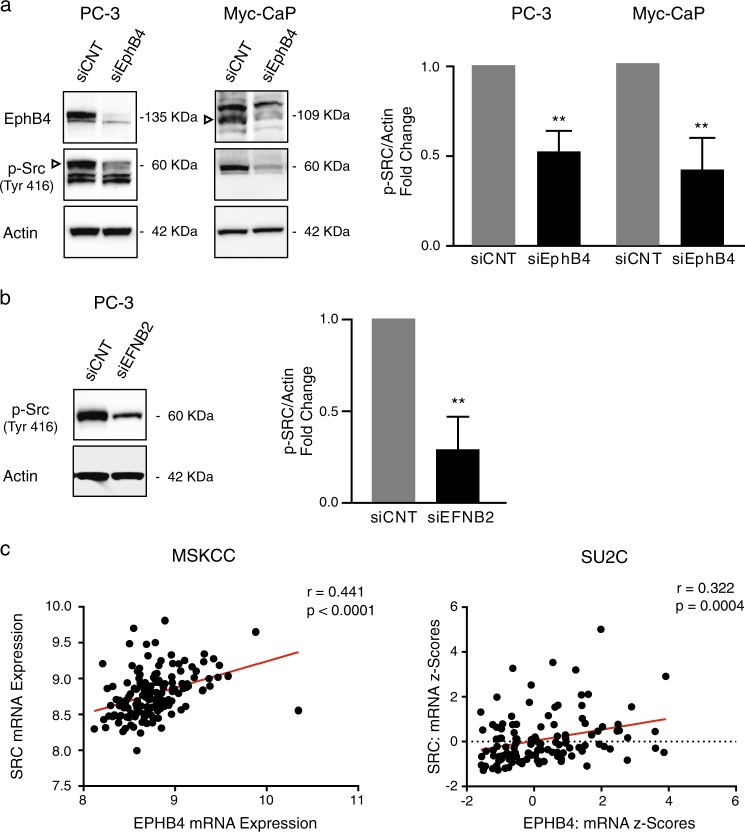
EPHB4 inhibition decreases phosphorylation of Src kinase. **a** Western blots with indicated antibodies on extracts from PC-3 and Myc-CaP cells transfected with control (siCNT) or with EphB4-specific siRNA (siEphB4) after 72 h. Data are presented as mean ± standard deviation (*n* = 3 or 4) from three different independent experiments. Open triangles indicate specific bands. **b** Western blots with indicated antibodies on extracts from PC-3 cells transfected with control (siCNT) or with EphrinB2-specific siRNA (siEFNB2) after 72 h. Data are presented as mean ± standard deviation (*n* = 3) from three different independent experiments. Statistical significance was analyzed by two-tailed Student’s *t*-test (treated group is compared to control group). *P < 0.05, **P < 0.01, ***P < 0.001, ****P < 0.0001. **c** EPHB4 correlation with SRC was analyzed in MKSC and S2UC dataset from Cbioportal.org

It has been shown that EPHB4 binding stimulates SRC kinase in ovarian carcinoma model^42^ and SRC kinase is known to play a role in regulating MAPK signaling pathway^43,44^. So, we hypothesized that the decrease in phosphorylation p38 MAPK upon EPHB4 inhibition could be explained by changes in the SRC activity. We therefore analyzed the activity of SRC via phosphorylation status after EPHB4 knockdown by siEPHB4–3 compared to siCNT in PC-3 and Myc-CaP cells. EPHB4 inhibition decreased phosphorylation of SRC at Tyr416 in both cell lines (Fig. 6a). We also observed decreased phosphorylation of SRC at Tyr416 after EFNB2 knockdown (Fig. 6b). In addition, we have also checked the available human prostate cancer patient dataset for EPHB4 correlation with SRC and found that EPHB4 was positively correlated with SRC kinase in prostate cancer patients (Fig. 6c). This result suggests that inhibition of EPHB4/EFNB2 decreases phosphorylation of P38 MAPK by reducing SRC kinase activity.

## Discussion

In this study, we show that targeting the receptor tyrosine kinase EPHB4 could sensitize prostate cancer to immune checkpoint blockade therapy. We describe for the first time to our knowledge that targeting EPHB4 induces ICD associated with ER stress, decreased glucose transporter and glucose uptake. EPHB4 is overexpressed in the majority of prostate cancers, particularly in advanced disease and is associated with a poor outcome. The possible mechanisms for increased expression of EPHB4 include loss of the tumor suppressors, PTEN and TP53, and activation of the PI3K pathway^19^. Thus, targeting EPHB4 could represent a viable modality for treating advanced prostate cancer.

Our results indicate that EPHB4 inhibition decreases prostate tumor cell proliferation and increases cell death in vitro. Apoptotic cell death has been documented to be immunologically silent and incapable of stimulating the immune response^45^. However, certain chemotherapeutic agents can induce cell death through the ICD mechanism^46^. We have found that EPHB4 knockdown or inhibition induces ICD by release of specific molecules belonging to the DAMP family, including exposure of calreticulin on the cell surface, release of HMGB1 into the extracellular space, and secretion of ATP. DAMPs are known to alert the immune system, resulting in the activation of the innate and adaptive immune response^47^. The process of ICD induction is well documented, and ER stress plays an important role in eliciting ICD^47^. ER stress is mainly accompanied by three sensors: PKR-like ER-localized eIF2α kinase (PERK), which is pathognomonic for ICD, activating transcription factor 6 (ATF6), and inositol-requiring 1 (IRE1)^23–25^. We demonstrate that EPHB4 knockdown or inhibition increased the phosphorylation of eIF2α and IRE1. ATP depletion can lead to ER stress since energy is required for protein folding, assembly^48^, and glycosylation^49^, and we found that inhibition of EPHB4 led to intracellular ATP depletion most likely due to decreased glycolysis as a result of reduced glucose uptake. The decreased glucose uptake after EPHB4 inhibition or depletion could be attributed to the decreased expression of the glucose transporter, GLUT3. Additionally, the reduced glycolysis in EPHB4 depleted cells can be explained by the decrease in MYC levels. MYC is known to enhance glycolysis, regulating glucose transporters and uptake in many cells^50–52^. Consistent with the observations in colon carcinoma cells^16^, we found that EPHB4 knockdown resulted in decreased MYC protein level and expression in prostate cancer cells. However, the underlying mechanism of EPHB4 mediated regulation of MYC has not yet been explored. The SRC family kinase has been reported to regulate the PI3K/AKT and ERK pathway in prostate cancer^53^ and also known to upregulate MYC level through cap-dependent translation via the ERK pathway in a breast cancer model^39^. It has been reported that SRC is activated by EPHB4 in ovarian carcinoma models suggesting the role of SRC kinase in regulation of MYC. Our data demonstrated that EPHB4 inhibition decreased the level of SRC and also positively correlated with SRC kinase in prostate cancer patients. p38 MAPK acts downstream to SRC kinase and is also known to phosphorylate 4EBP1^40^. We discovered that EPHB4/EFNB2 inhibition decreased the phosphorylation of p38 MAPK mediated by SRC, resulting in repression of cap-dependent translation machinery by decreased phosphorylation of 4EBP1. Of note, although we could not completely rule out the possibility of off-target effects of EPHB4 inhibitor (NVP-BHG712) in our experiments, this concern is minimized due to the observation that EPHB4 siRNA as well as EFNB2 siRNA gave similar results to the chemical inhibitor.

**Fig. 7:**
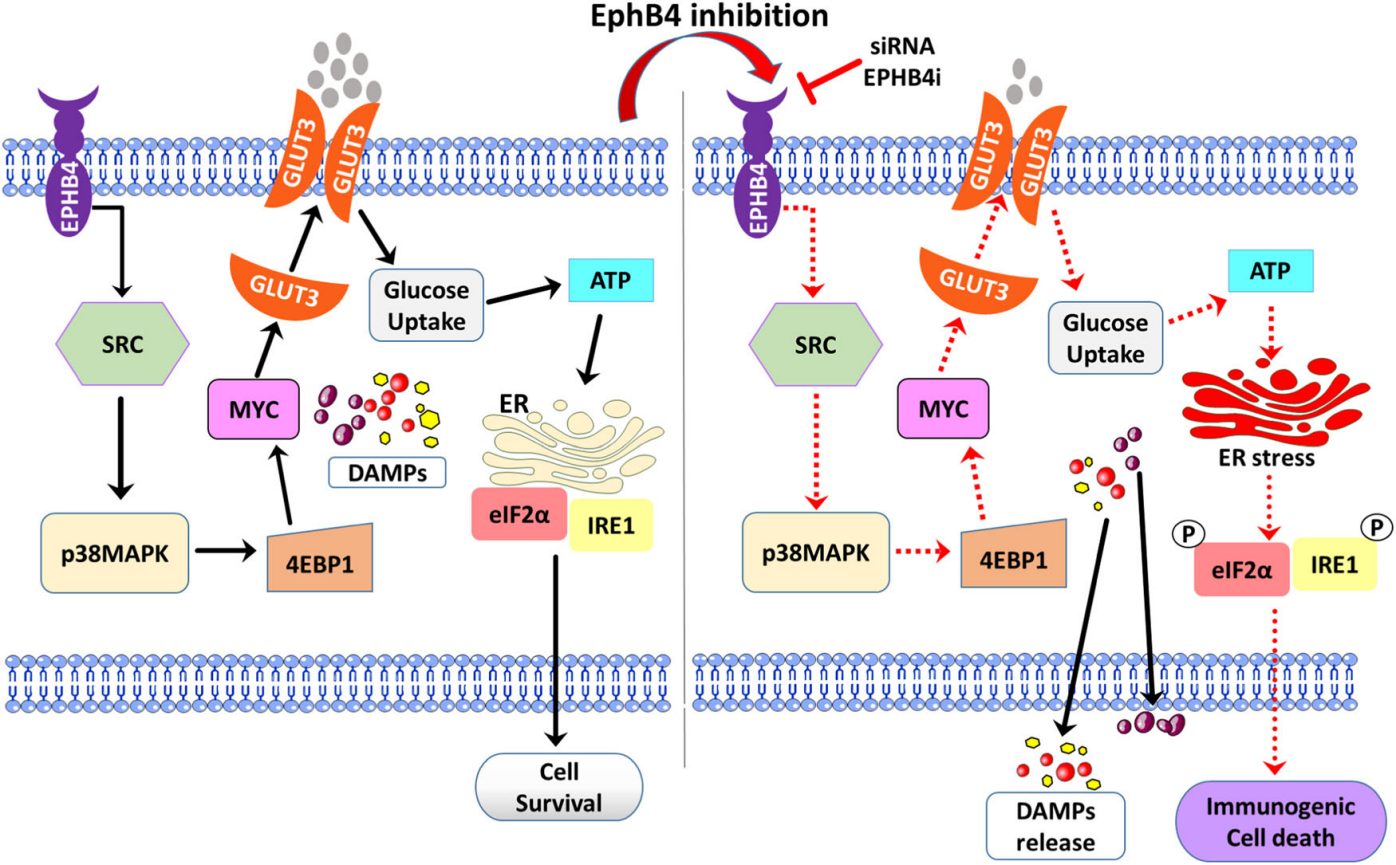
Proposed EPHB4 signaling pathway in prostate cancer cells. EPHB4 promote signaling through SRC-P38MAPK-4EBP1 pathway that leads to activation of MYC and MYC mediated metabolism for cell growth and survival in prostate cancer cells. DAMPs are located intracellularly and hence are unrecognizable by the immune system. EPHB4 inhibition decreases cap-dependent translation of MYC by suppressing SRC-p38MAPK-4EBP1 pathway. Suppression of SRC-p38MAPK-4EBP1 leads to a decrease in GLUT3 expression, which in turn leads to a reduced uptake of glucose and a drop in the ATP levels. This effect induces ER stress and tumor cell death characterized by ICD, including phosphorylation of eIF2α, increased cell surface calreticulin levels, and release of HMGB1 and ATP

In summary, our study has identified a novel role for the receptor tyrosine kinase EPHB4 signaling pathway in regulating prostate cancer cell survival that can be exploited for targeted therapy. Specifically, EPHB4 inhibition impairs tumor cell metabolism though changes in glucose transporter and glucose uptake, resulting in ER stress and immunogenic cell death. The impairment of tumor cell metabolism arises due to MYC deactivation as a result of the SRC-p38 MAPK-4EBP1 signaling (Fig. 7). Our data demonstrate that induction of ICD that triggers the release of immune-modulatory danger signals that are known to stimulate an anti-tumor immune response. Therefore, this studies demonstrates that EPHB4 targeted therapy may be capable of changing the prostate tumor microenvironment from “cold to “hot’, thereby increase sensitivity to immune checkpoint inhibitor therapy.

## Materials and methods

### Cell culture

Human prostate cancer cell lines PC-3, 22Rv1, and LNCaP were from ATCC and mouse Myc-CaP cells were the kind gift of Charles Sawyers (Memorial Sloan-Kettering Cancer Centre). Myc-CaP; Pten-KO cells are Myc-CaP cells with deletion of *Pten* by CRISPR/Cas9 and have been described^54^. Cells were authenticated and verified to be mycoplasma free. Cells were maintained at 37 °C in a humidified incubator and 5% CO_2_ atmosphere in RPMI 1640 medium (Gibco, Thermofisher Scientific) supplemented with 10% heat inactivated fetal bovine serum (FBS; Corning), 1% of Penicillin-Streptomycin (10,000 U/ml; Life Technologies). For EPHB4 inhibition, inhibitor NVP-BHG712 (Selleck Chemicals) was dissolved in DMSO (Sigma). Cell viability was assessed by CellTiter 96 AQueous One Solution Cell Proliferation Assay (Promega) and Cell Counting Kit-8 (Dojindo Molecular Technologies). Cells were seeded at 5000 cells/well in 96-well plates (Corning) and allowed to adhere overnight. Cells were then transiently transfected with siRNA specific for *EPHB4* or non-targeting control (Dharmacon). For EFNB2 knockdown, cells were transiently transfected with siRNA specific for *EFNB2* or non-targeting control (Dharmacon). Absorbance was measured at 72 h at 490 nm in a microplate reader. For apoptosis, Caspase-3/7 activities were evaluated by Caspase-Glo 3/7 Assay (Promega) according to the manufacturer’s recommendation.

### Organoid culture

NCI-H660 cells (ATCC) were seeded at 5000 cells/well density in ultra-low attachment 96-well plates (Corning) and cultured in Hepatocyte growth media (Corning) supplemented with 10 ng/ml epidermal growth factor (Corning), 5% heat inactivated charcoal stripped FBS, 1X Glutamax (Gibco), 5% Matrigel (BD Biosciences), 10 µM ROCK inhibitor (Y-27632, STEMCELL Technologies), and 1X Gentamicin/Amphotericin (Lonza), as described previously^55^. At day 8, organoids were treated with NVP-BHG 712 (EPHB4i) from Selleck Chemicals or DMSO (Veh.), and viability was assessed at day 15 as per manufacturer’s protocol (Cell Titer Glo-3D viability assay, Promega).

### RNA interference

Cells were transiently transfected with 25 nM of human siGENOME EphB4 siRNA (Set of four *EPHB4* siRNA, Dharmacon Catalog no. # MU-003124–02–0002) or mouse siGENOME EphB4 (Dharmacon Catalog no. # MU-06046901–0002) or Non-targeted siRNA (Dharmacon Catalog no. D-001210–01–05), human siGENOME *EFNB2* siRNA, Dharmacon Catalog no. # M-003659–02–0005), siGENOME Human *MYC* siRNA, Dharmacon catalog no. M-003282–07–0005 and DharmaFECT transfection reagent (Dharmacon), according to the manufacturer’s protocol for 72 h. After 20 min of incubation, the mixture was added to the suspended cells and these were plated in dishes for each assay. Cells were analyzed for all experiments after 72 h.

### Immunogenic cell death assay

Myc-CaP cells were transfected with siEPHB4 or siCNT, or treated with EphB4 inhibitor for 72 h. After the indicated times, supernatants were collected and cell counts performed for quantifying secreted ATP (Bioluminescent Assay Kit, Sigma) and high-mobility group protein B1 (HMGB1; Elisa, Techan Trading). For detection of surface Calreticulin, cells were incubated with rabbit anti-Calreticulin (1:1000, Abcam ab2907) for 60 min and then incubated with Alexa Fluor 488 anti-rabbit secondary antibody (Invitrogen A11008 1 µg/ml) and analyzed by flow cytometry.

### qRT-PCR

Total RNA extraction from PC-3 transfected cells were performed using the TRIzol reagent (Life Technologies, Rockville, MD), according to the manufacturer’s instructions. The reverse transcriptase polymerase chain reaction (RT-PCR) was performed using Moloney murine leukemia virus reverse transcriptase (MMLV; Invitrogen, Carlsbad, CA) as per manufacturer’s instructions. qRT-PCR was performed using PowerUp SYBR Green Master Mix (Applied Biosystems) and following primers: EFNB2 (F: TATGCAGAACTGCGATTTCCAA, R: TGGGTATAGTACCAGTCCTTGTC) or the housekeeping gene 18S rRNA (F: GTAACCCGTTGAACCCCATT, R: CCATCCAATCGGTAGTAGCG) (Integrated DNA Technologies).

### Western blot

This was performed as described^56,57^. The following primary antibodies were used: EPHB4 (D1C7N) (1: 1000, Cell Signaling #14960), EPHB4 (1:500, Abcam #ab73259), phospho-eIF2α S51 (1:1000, Cell Signaling #9721 S), eiF2α (1:000, Cell Signaling #9722), phospho-IRE1(S724) (1:1000, Abcam #ab48187**)**, c-Myc (Y69) (1:5000, Abcam #ab32072), GLUT3 (1:5000, Abcam #ab191071), Calreticulin (1:400, Abcam #ab2907), HMGB1 (Abcam #ab18256), Actin (1:10000, Cell Signaling #5125), GAPDH (1:5000, Cell Signaling #3683), Phospho-Src Family Tyr416 (1:1000, Cell Signaling #2101), Phospho-p38 MAPK Thr180/Tyr182 (1:1000, Cell Signaling #9211), and Phospho-4EBP1 Thr37/46 (236B4) (1:1000, Cell Signaling #2855). Following incubation with horseradish peroxidase-conjugated goat anti-rabbit/mouse (1:5000, Biorad) or goat anti-rat (1:5000, Santa Cruz) for 1 h, the binding of secondary antibodies were detected with the Supersignal West Femto Maximum Sensitivity Substrate detection system (Pierce) followed by visualization. Quantification analyses were performed by Biorad ChemiDoc Imager and Bio-Rad Image Lab software.

### Immunofluorescence

Cells were transfected with siEPHB4 or siCNT, after transfection cells were plated in 4-well chamber slides for 72 h, slides were incubated with primary antibody c-Myc and followed by secondary antibody labeled with Alexa Fluor 488 anti-rabbit (Thermo Scientific # A11008). Slides were mounted with ProLong Gold Antifade reagent (Invitrogen/Molecular Probes #P36961). Immunofluorescence images were visualized using fluorescent microscope or Leica A1R spectral confocal microscope.

### Glucose uptake assay

Cells were transfected with siEphB4 or siCNT in 96-well plates. Glucose uptake was measured 72 h post-transfection using Glucose Uptake-Glo™ Assay (Promega) according to the manufacturer’s instructions.

### Intracellular ATP assay

Cells were transfected with siRNA or treated with EphB4 inhibitor in 96-well plates. After 72 h, ATP level was measured using the ATPlite Luminescence Assay System from Perkin Elmer as described^58^.

### Human Prostate cancer data

Gene expression data were downloaded from the NCBI Geo for the following datasets: Tomlins Prostate cancer dataset [GSE6099; Normal tissues (Adjacent Normal Epithelium, BPH; *n* = 22), Prostate cancers (PIN, Primary Prostate cancer, Metastatic cancer samples; *n* = 60)], Wallace Prostate cancer dataset [GSE6956; surrounding non-tumor tissues (*n* = 18), prostate cancers (*n* = 69)], Grasso Prostate cancer dataset [GSE35988; benign prostate tissues (*n* = 28), localized primary cancers (*n* = 59), metastatic CRPC (*n* = 35)], and Roudier prostate cancer dataset [GSE74367; Primary prostate cancer (*n* = 11), CRPC metastases (*n* = 45)]^59–62^. *P*-values were determined by Welsh’s *t*-test. For survival analysis, data were downloaded from NCBI Geo (GEO accession no. GSE70769)^21^ and cBioPortal (TCGA provisional). Patient groups were classified as high EPHB4 and low EPHB4 expression by the 75th percentile for the Ross-Adams dataset and the 90th percentile for the TCGA study population. Patient survivals were assessed by the Kaplan–Meier method. *P*-values were determined by log-rank test.

For Correlation analysis, the gene expression of EPHB4 and SRC were analyzed using Spearman’s rank correlation. Data used for this analysis was from the MSKCC dataset (*n* = 150) and SU2C dataset (*n* = 118) downloaded from cbioportal.org.

### Statistical analysis

All statistical analyses were performed in GraphPad Prism 7 software. The number of technical replicates, biological replicates, and independent experiments performed are indicated in the figure legends. Statistical analyses were by a two-tailed Student’s *t*-test. Data are presented as mean ± standard error of the mean (S.E.M.), for analyses, results were considered statistically significant with *P* < 0.05, *P < 0.05, **P < 0.01, ***P < 0.001, and ****P < 0.0001.
